# Optimized DNA-based identification of *Toxocara* spp. eggs in soil and sand samples

**DOI:** 10.1186/s13071-021-04904-1

**Published:** 2021-08-26

**Authors:** Wojciech Jarosz, Jean-Francois Durant, Leonid Mwana Wa Bene Irenge, Renata Fogt-Wyrwas, Hanna Mizgajska-Wiktor, Jean-Luc Gala

**Affiliations:** 1 Department of Biology and Anatomy, Faculty of Health Sciences, Poznań University of Physical Education, Królowej Jadwigi 27/39, 61-871 Poznan, Poland; 2 Center for Applied Molecular Technologies, Institute of Clinical and Experimental Research, Université Catholique de Louvain, Tour Claude Bernard, Avenue Hippocrate 54-55, 1st Floor, B1.54.01, 1200 Brussels, Belgium; 3 Defense Laboratories Department, ACOS Ops & Trg, Belgian Armed Forces, Martelarenstraat, 181, 1800 Peutie, Belgium

**Keywords:** *Toxocara canis*, *Toxocara cati*, Helminth eggs, Limit of detection, Soil, Sand, Inhibitors, DNA extraction, qPCR, Clean-up

## Abstract

**Background:**

*Toxocara canis* and *Toxocara cati* are globally distributed roundworms and causative agents of human toxocariasis, via ingestion of *Toxocara* eggs. Control of *Toxocara* infections is constrained by a lack of sensitive methods for screening of animal faeces and environmental samples potentially contaminated by *Toxocara* eggs. In this work, a pre-analytical method for efficient extraction of DNA from *Toxocara* eggs in environmental samples was set up using our previously validated *T*. *canis*- and *T*. *cati*-specific quantitative real-time polymerase chain reaction (qPCR). For this purpose, the influence of different methods for egg lysis, DNA extraction and purification for removal of PCR inhibitors were assessed on environmental samples.

**Methods:**

To select the best egg disruption method, six protocols were compared on pure *T*. *canis* egg suspensions, including enzymatic lysis and thermal or mechanical disruption. Based on the selected best method, an analytical workflow was set up to compare two DNA extraction methods (FastDNA™ SPIN Kit for Soil versus DNeasy^®^ PowerMax^®^ Soil Kit) with an optional dilution and/or clean-up (Agencourt^®^ AMPure^®^) step. This workflow was evaluated on 10-g soil and 10-g sand samples spiked with egg suspensions of *T*. *canis* (tenfold dilutions of 10^4^ eggs in triplicate). The capacity of the different methods, used alone or in combination, to increase the ratio of positive tests was assessed. The resulting optimal workflow for processing spiked soil samples was then tested on environmental soil samples and compared with the conventional flotation-centrifugation and microscopic examination of *Toxocara* eggs.

**Results:**

The most effective DNA extraction method for *Toxocara* eggs in soil samples consisted in the combination of mechanical lysis of eggs using beads, followed by DNA extraction with the DNeasy^®^ PowerMax^®^ Soil Kit, and completed with an additional DNA clean-up step with AMPure^®^ beads and a sample DNA dilution (1:10). This workflow exhibited a limit of detection of 4 and 46 *T.* *canis* eggs in 10-g sand and 10-g soil samples, respectively.

**Conclusions:**

The pre-analytical flow process developed here combined with qPCR represents an improved, potentially automatable, and cost-effective method for the surveillance of *Toxocara* contamination in the environment.

**Graphical Abstract:**

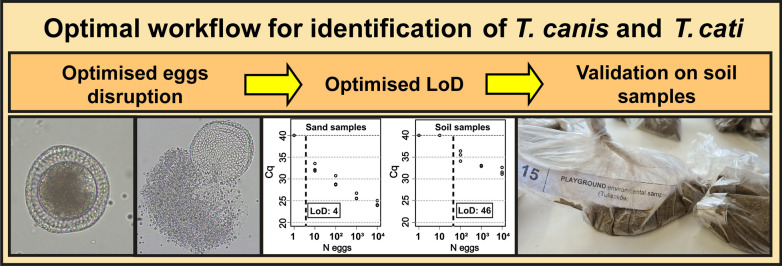

**Supplementary Information:**

The online version contains supplementary material available at 10.1186/s13071-021-04904-1.

## Background

*Toxocara canis* and *T*. *cati* are parasitic roundworms that are causative agents of toxocariasis, a widespread neglected zoonotic disease [1–3] that afflicts millions of people worldwide [4]. The disease is transmitted to humans through ingestion of soil contaminated with *T*. *canis* eggs from faeces of dogs and *T*. *cati* from faeces of cats [5]. Determining the extent of environmental contamination with *Toxocara* spp. eggs, among which the relative importance of different definitive hosts as sources of ova, is considered one of the knowledge gaps in the epidemiology of *Toxocara* [2].

Despite the development of sensitive quantitative real-time polymerase chain reaction (qPCR) assays for the detection of *T*. *canis* and *T*. *cati* eggs [6], low numbers of *Toxocara* eggs in environmental samples still constitute a shortcoming in the control of toxocariasis [7]. This has resulted in low sensitivity of DNA extraction methods available for *Toxocara* eggs in soil samples. Although several protocols for extracting helminth DNA from environmental samples have been developed [8], they have not displayed high sensitivity for *Toxocara* eggs in soil samples [9]. An experimental method combining *Toxocara* egg enrichment by the flotation technique and subsequent DNA extraction from soil samples spiked with *Toxocara* eggs resulted in a *Toxocara* detection rate of 41.7% in 10 g of soil samples spiked with 10 eggs, and only 8.3% for samples spiked with one egg [9]. These results underscore the need for alternative methods which provide sensitive detection of *Toxocara* eggs in soil samples. The aim of this study was to optimize and validate DNA extraction of *T*. *canis* eggs from soil and sand samples. This entailed the assessment of (i) efficient *Toxocara* egg disruption, (ii) efficient DNA extraction and (iii) removal of PCR inhibitors which might be present in DNA solutions. The optimisation of these analytical steps was expected to improve PCR-based detection of *Toxocara* in environmental samples.

## Methods

### Rationale of the study

The rationale supporting this study was a stepwise selection of an optimised analytical protocol exhibiting the highest qPCR positivity rate for the presence of *Toxocara* eggs in sand and soil samples spiked with *T*. *canis* eggs. A series of methods and commercially available extraction and DNA purification kits were used, assessed and compared following a three-step procedure as detailed herein (Additional file 1: Figure S1). The ultimate goal was to use the best protocol on collected (*n* = 40) environmental soil samples where the potential detrimental effect of qPCR inhibitors on the sensitivity of *Toxocara* spp. egg detection is well known, and to compare it to the standard method, which is the conventional flotation-microscopic observation.

### Soil sample collection

Egg-spiking experiments were carried out in presumably *Toxocara*-free clean sand and soil samples—commercial sand and soil from a backyard in Poznań, Poland (52° 24′ 52″ N, 16° 55′ 16″ E) without any history of dog, cat or fox presence and doubly confirmed negative for *Toxocara* spp. eggs with the flotation method described below. Environmental soil samples of 250 g (*n* = 40) were collected according to a systematic unaligned sampling method, from the upper soil layer (3 cm) [10] in and around Tuliszków, Poland, (52° 04′ 35″ N, 18° 17′ 37″ E), also covering nearby rural villages. The sites included playgrounds (*n* = 20) and backyards close to households (*n* = 20). After collection, the samples were dried for 24–48 h and sifted through a 2 mm sieve to remove stones and larger organic particles.

### Egg stock solution and serial dilutions

*Toxocaracanis* fertilized eggs were isolated from uteri of adult female worms and suspended in nuclease-free molecular biology-grade water (HyClone™ HyPure). Four aliquots of 5 µl of the unembryonated egg suspensions were observed under the light microscope (100-fold magnification). The number of eggs was calculated per field and then reported as the grand mean of the eight squares ± standard deviation (SD). Serial dilutions (i.e., 10^4^, 10^3^, 10^2^, 10, 1) eggs were prepared in DNA/RNA-free water.

### Eggshell disruption methods

Six methods for disruption of *T*. *canis* eggs (1, 10, 10^2^ and 10^3^ egg suspensions) were compared: (i) enzymatic lysis with proteinase K (PK) [incubation of egg solution with 0.2 unit of proteinase K in 40 µl solution containing 10% (w/v) of SDS at 56 °C under agitation at 800 rpm for 2 h using a thermomixer (Eppendorf, Hamburg, Germany)]; (ii) thermal disruption (TD) (5 freeze–thaw cycles: 3 min of freezing in liquid nitrogen, followed by 3 min of thawing in boiling water under agitation at 800 rpm in a thermomixer); (iii) mechanical disruption of eggs using FastPrep^®^ tubes containing the lysing matrix A beads (FPA) (MP Biomedicals, Santa Ana, CA, USA) under shaking at 6 m/s for 40 s in a FastPrep-24 homogenizer (three cycles); (iv) the same protocol as the previous but using lysing matrix D beads (FPD) instead; (v) TD followed by FPD (TD-FPD); and (vi) TD-FPD followed by PK (TD–FPD-PK). Following the disruption step, DNA was extracted using the NucliSENS^®^ MiniMag^®^ Kit (bioMérieux, Boxtel, Netherlands) according to the manufacturer’s protocol. DNA solutions were stored at −20 °C until use.

### DNA extraction from sand and soil samples using commercial kits

*Toxocara*-free soil and sand samples (10 g) were spiked in triplicates with serial tenfold dilutions (10^4^ to 1) of *T*. *canis* eggs. For the selection of the more efficient DNA extraction method in sand and soil samples, two kits representing the best disruption methods (i.e., DNeasy^®^ PowerMax^®^ Soil Kit, Qiagen, Hilden, Germany, and the FastDNA™ SPIN Kit for Soil, MP Biomedicals, Santa Ana, CA, USA) were used according to the manufacturer’s instructions. The kit producing the best result was then used for DNA extraction from soil samples (*n* = 40) collected in a rural area around Tuliszków in Poland for detection of *T*. *canis*/*T*. *cati* eggs using our optimised *T*. *canis*- and *T*. *cati*-specific duplex qPCR [6].

### DNA purification (clean-up step)

The impact of a clean-up step for removal of PCR inhibitors was assessed by performing duplex qPCR for *T*. *canis* and *T*. *cati* on DNA before and after a clean-up step. A magnetic beads DNA method (Agencourt^®^ AMPure^®^, Beckman Coulter, MA, USA) was used for DNA clean-up (i.e., for removal of PCR inhibitors) in all samples. Briefly, 1.8 volume of AMPure^®^ beads was added to one volume of extracted DNA. The DNA-beads complex was placed on a magnetic stand and the solution was discarded. The DNA was then washed 2× with 70% ethanol. DNA was eluted in one volume of DNA/RNA-free water. All purified DNA solutions were kept at −20 °C until use. DNA quantity was measured by nanodrop but the concentration values were under the dynamic range of the instrument; consequently *Toxocara*-specific qPCR—as described below—was used to quantify *Toxocara* DNA from each DNA extracted sample.

### Quantitative real-time PCR

Specific quantitative real-time polymerase chain reaction (qPCR) targeting *T*. *canis* (for samples spiked with eggs) or *T*. *canis* and *T*. *cati* (for environmental samples) was carried out in triplicate on extracted undiluted and diluted (1:10) DNA samples according to the procedure previously described by Durant et al. [6] on a CFX96 thermocycler (Bio-Rad, Hercules, CA, USA). An Ascaridoidea-generic qPCR was used as internal quality control. The qPCR results were expressed as quantification cycle (Cq). Cq values were flagged as “undetermined” by the thermocycler software when reaching 40 cycles, and this threshold was defined as a negative result.

### Flotation method and microscopic observation

Environmental samples were processed using the flotation-centrifugation method [11] using the Sheather’s sugar solution specific gravity of 1.27. Two coverslips were used to recover eggs from each sample, examined under light microscopy for counting of *T*. *canis* eggs. Recovered eggs were characterized based on their size, the thickness of eggshells, transparency and visibility of semi-circular cavities on their surfaces.

### Statistical analysis

For each of the egg disruption methods tested, extraction yield was calculated on the basis of the qPCR Cq values. When only one PCR reaction from the three replicates led to a missing value, this missing value was excluded from downstream analysis, as described and recommended in a recent survey on qPCR data analysis [12]. A calibration curve was built for each method and the limit of detection (LoD) [13] was calculated at the intersection between the limit of blank (LoB) and the prediction interval of the calibration curve. In the current study, the LoD is therefore the lowest number of eggs likely to be reliably distinguished from the LoB and at which detection is feasible. To determine the concordance between results obtained with tested extraction methods, Cohen’s kappa statistic was calculated in field samples. All statistical analyses were performed using R 3.4 and SPSS^®^ Statistics software (IBM Corporation, Armonk, NY, USA).

## Results

### Dilution series of egg disruption methods

The qPCR Cq values obtained with each *Toxocara* egg disruption method are shown in Additional file 2: Figure S2. TD, FPD and TD–FPD-PK (LoD = 7 eggs) were the three disruption methods displaying the lowest LoD with the best yield. FPD was selected over TD–FPD-PK as it is handy and easy to use in the field. Accordingly, DNA extraction kits which include a mechanical egg disruption step were selected for assessment of the presence of *T*. *canis* in sand and soil samples spiked by serial dilutions of *T*. *canis* eggs.

### DNA extraction from sand and soil samples using commercial kits

**Fig. 1:**
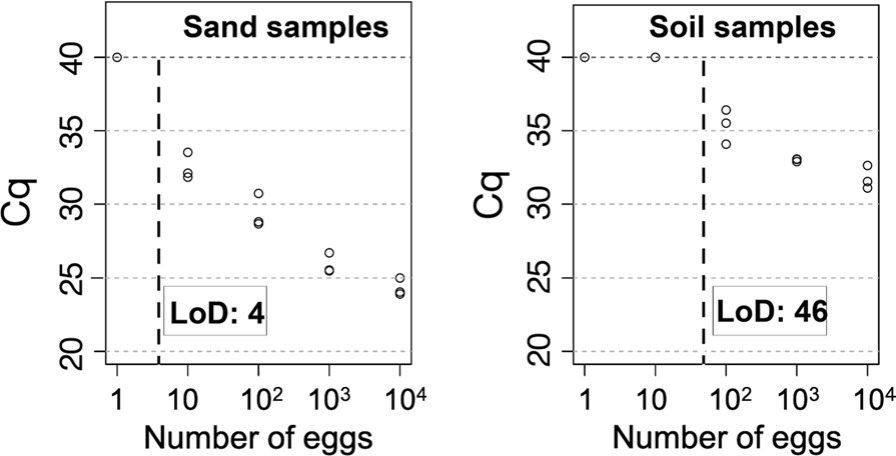
DNA extraction efficiency on serial dilutions of *Toxocara canis* eggs spiked in 10-g sand and 10-g soil samples. This figure shows standard curves expressing Cq values from sand and soil samples spiked with tenfold serial dilutions (10^4^ to 1) of *T*. *canis* eggs. DNA extraction was performed using the DNeasy^®^ Power Max^®^ Soil Kit. DNA from sand samples was used unpurified and undiluted, whereas DNA from soil samples was purified and diluted 1:10

qPCR Cq values from sand and soil samples spiked with serial tenfold dilutions of *T*. *canis* eggs (from and 10^4^ to 1) and processed with the DNeasy^®^ PowerMax^®^ Soil Kit and with the FastDNA™ SPIN Kit for Soil are shown in Table 1. The LoD was 4 and 46 *T. canis* eggs in 10 g of sand and soil samples, respectively, with a probability of 95% for both LoDs (Fig. 1). Typical amplification curves are shown in Additional file 3: Figure S3.

**Table 1:** DNA extraction efficiency on tenfold serial dilutions of *Toxocara canis* eggs (10^4^ to 1) spiked in 10-g sand and 10 g-soil samples, by comparing two extraction kits, with or without an additional DNA purification and/or DNA dilution (1:10) step

Commercial kits	Sample type	Amount of spiked eggs	qPCR results quantification cycle (Cq)			
			No clean-up step		AMPure^®^ clean-up step	
			Undiluted DNA	Diluted DNA (1:10)	Undiluted DNA	Diluted DNA (1:10)
DNeasy^®^ Power Max^®^ Soil Kit	Sand	1	Negative	Negative	Negative	Negative
		10	32.49 ± 0.82	35.40 ± 1.26	34.25 ± 1.51	Negative
		100	29,39 ± 1,01	32.54 ± 1.68	32.58 ± 1.24	37.57 ± 0.24
		1000	25,91 ± 0,61	29.11 ± 0.64	29.08 ± 1.02	34.04 ± 0.65
		10,000	24.32 ± 0,53	26.61 ± 0.22	24.02 ± 0.14	27.45 ± 0.09
	Soil	1	Negative	Negative	Negative	Negative
		10	Negative	Negative	Negative	Negative
		100	Negative	Negative	Negative	35.34 ± 1.26
		1000	Negative	Negative	Negative	33.01 ± 0.17
		10,000	Negative	Negative	29.23 ± 1.32	31.76 ± 0.73
FastDNA™ SPIN Kit for Soil	Sand	1	Negative	Negative	Negative	Negative
		10	Negative	Negative	Negative	Negative
		100	Negative	Negative	Negative	Negative
		1000	Negative	Negative	Negative	Negative
		10,000	34.69 ± 0.23	37.66 ± 0.24	37.14 ± 1.11	Negative
	Soil	1	Negative	Negative	Negative	Negative
		10	Negative	Negative	Negative	Negative
		100	Negative	Negative	Negative	37.17 ± 0.59
		1000	Negative	Negative	Negative	35.77 ± 0.97
		10,000	Negative	Negative	Negative	30.03 ± 1.00

### Results without clean-up procedure

Results obtained with both DNA extraction kits for soil samples are in line with a higher level of qPCR inhibition in soil than in sand samples, and with higher efficacy of the DNeasy^®^ PowerMax^®^ Soil Kit for removing qPCR inhibitors. However, this removal effect was only visible with sand samples, as no positive signal was obtained with any soil sample, irrespective of the egg concentration. Diluting DNA weakened the positive signals from sand samples while failing to improve results obtained with soil samples, in line with the persistence of inhibitors in the latter samples.

### Results comparison after clean-up procedure

The clean-up procedure confirmed its usefulness for removing soil inhibitors in soil samples. The DNeasy^®^ PowerMax^®^ Soil Kit indeed generated a positive signal on soil samples but only at the highest egg concentration. On the contrary, this step had a detrimental effect on strength of positive signals obtained with sand samples. Diluting DNA further decreased the positive signals from sand samples. Combining a clean-up and DNA dilution step on soil samples generated positive signals with both DNA extraction kits, whereas signals obtained with the DNeasy^®^ PowerMax^®^ Soil Kit were higher at low (10^2^ and 10^3^) than at high (10^4^) egg concentration.

### Environmental sample analyses

A total of 40 environmental samples collected from backyards and playgrounds were assessed for the presence of *Toxocara* eggs through the processing of 10 g of soil (from a total of 250 g) using the most effective protocol as identified from the above comparison of methods (i.e., DNeasy^®^ PowerMax^®^ Soil Kit, AMPure^®^ clean-up step and DNA dilution 1:10). In parallel, 40-g soil samples were processed through conventional microscopic examination after an enrichment (flotation) step. qPCR and microscopic observation results are summarized in Additional file 4: Table S1.

Altogether, the duplex-specific qPCR was positive for *T*. *canis* in 8/40 samples while no signal relating to the presence of *T*. *cati* was generated and the Ascaridoidea*-*generic qPCR was consistently positive in all samples outlining the presence of Ascaridoidea DNA in all environmental samples examined.

Overall, there was moderate agreement between light microscopic observation of enriched soil samples after flotation, with Cohen's κ = 0.423 (*P* < 0.005). However, the molecular assay (i.e., DNA extraction using the DNeasy^®^ PowerMax^®^ Soil Kit plus AMPure^®^ clean-up and duplex qPCR) enabled the detection of more positive samples when compared to light microscopy observation on flotation-enriched samples. Additional file 5: Figure S4 gives a global overview of the successive steps and main outcomes.

## Discussion

Soil is considered the primary source of *Toxocara* spp. transmission to humans, especially children [14]. Soil contamination by eggs of *Toxocara* spp. occurs with faeces from infected dogs and cats. Furthermore, the survival of *Toxocara* eggs from dogs and cats in the environment for months or years is an additional risk factor for contamination [15]. However, detection of *Toxocara* eggs in soil samples in the frame of environmental surveillance programs is hampered by the lack of reliable and sensitive analytical methods. While enrichment by flotation and subsequent light microscopic examination remains the reference method, this method is difficult to perform in the field, is time-consuming, and requires experience for light microscopy and parasitological diagnosis.

For maximising the disruption of the thick wall of *Toxocara* eggs, different DNA extraction methods were compared, as a first step. A bead-beating step was shown to be preferable to enzymatic and thermal lysis. The next step was then to use DNA extraction kits including beads for processing sand and soil samples spiked with serial dilutions of eggs. Results were assessed with and without a clean-up step for removal of PCR inhibitors. DNeasy^®^ PowerMax^®^ Soil Kit gave the best results in terms of Cq values with sand samples, but no soil sample was positive without a clean-up step. The AMPure^®^ beads purification was compared with the PowerClean Pro Cleanup Kit (Qiagen, Hilden, Germany), and displayed similar efficiency (data not shown). The AMPure^®^ clean-up was favoured, as it is compatible with the prospect of automation of the whole analytical processing. This additional step did not improve the qPCR signals obtained with sand samples but appeared to be necessary to generate a positive qPCR signal with soil samples, albeit only at the highest egg concentration. For soil samples, an additional dilution step was necessary. Using the best analytical workflow enabled us to achieve sensitivity of 4 and 46 eggs in 10 g of sand and soil, respectively. To the best of our knowledge, this is an improvement compared to previously published methods [6, 9]. A method combining flotation and qPCR achieved a detection threshold of 10 eggs for the flatworm *Echinococcus multilocularis* per 10 g of soil but failed to reach a similar figure for *Toxocara* eggs [9]. Although the causes behind this low sensitivity for detection of *Toxocara* DNA are not known, they might be associated with the use of methods which do not emphasise the mandatory disruption of eggs prior to DNA extraction. By improving this step, we have been able to achieve sensitivity comparable to that observed with other roundworms. However, current data show that total removal of PCR inhibitors from soil samples cannot be guaranteed, as pointed out by other authors [16]. This contradicts manufacturers’ claims that their extraction kits are efficient in removing PCR inhibitors from soil samples.

A third step was to then analyse a series of environmental soil samples collected from backyards or playgrounds, using the most efficient analytical workflow as previously defined on spiked soil samples. Results were compared with the conventional flotation-microscopic evaluation. Interestingly enough, the latter gave a lower positivity rate. When considering the potential hurdle of non-homogeneous distribution of *Toxocara* eggs, it is worth noting that samples 7 and 14, which came from the same playground, did not display similar qPCR results. A solution might reside in sampling more areas. As already commented above, PCR inhibitors as the cause of a PCR negative result cannot totally be ruled out. The same observation applies to samples 31, 32 and 40, which came from the same backyard. Of note, the alleged improved sensitivity on detection of helminths such as *Echinococcus multilocularis* was achieved by combining an enrichment (flotation) step with qPCR [9]. This renders the method cumbersome and time-consuming, two features which preclude the use of the method in the field for screening of hundreds of environmental samples. Another lingering question, not assessed in the current study, is whether positive qPCR results can be taken at value face as proof of soil contamination, given that dead *Toxocara* eggs can be associated with positive qPCR signals without also being able to cause toxocariasis when ingested by humans. There is no definitive answer to this question, but the long-term survival of *Toxocara* eggs in the environment [15] and the probability that viable eggs can be mistaken for dead eggs upon light microscopic observation should prompt us to consider qPCR positive as a reliable surrogate marker of soil contamination with *Toxocara* eggs.

Finally, we assessed the cost of the optimized workflow to process a batch of 10 samples with the optimal DNA extraction kit (~ 64€/sample, details in Additional file 6: Table S2). The optimized DNA-based analysis remains more expensive than the handmade classical method. However, it can already be anticipated that the automated processing of large numbers of samples will make DNA analysis less time-consuming, labour-intensive and expensive than manual sample processing, while also significantly increasing the egg detection positivity rate.

## Conclusions

In conclusion, the sample processing developed here, which combines a mechanical disruption of *Toxocara* eggs, DNA extraction using the DNeasy^®^ PowerMax^®^ Soil Kit and a subsequent DNA clean-up for removal of PCR inhibitors, improves substantially the qPCR detection of *Toxocara* eggs in soil samples. While the analytical cost per batch of samples is higher than the conventional microscopic examination, current automated analytical procedures have the potential to substantially decrease the cost per sample, and to simplify and speed up the analysis, allowing timely results to be delivered within a short period. Combined with a significantly higher positivity rate, these advantages are worth considering when a large number of analyses need to be performed rapidly in field conditions.

## Supplementary Information

**Additional file 1: Figure S1.** Stepwise optimisation of the analytical workflow for improving the DNA-based identification of *Toxocara* spp. eggs.

**Additional file 2: Figure S2.** Comparison of six egg disruption methods and a control (no egg disruption): disruption efficiency is expressed by qPCR Cq values.

**Additional file 3: Figure S3.** Examples of qPCR amplification curves according to sample types (sand, soil). qPCR amplification curves obtained after extraction of 10^4^ eggs spiked in sand (**a**, **b**) and soil samples (**c**, **d**) using the DNeasy^®^ PowerMax^®^ Soil kit. **a**, **c** qPCR curves generated without clean-up. **b**, **d** qPCR curves generated after purification (AMPure^®^). The qPCR amplification curves obtained with the *Toxocara canis* DNA positive control, non-template control (NTC), undiluted DNA and diluted (1:10) DNA are represented in green, black, blue and red, respectively.

**Additional file 4: Table S1.** qPCR Cq values (mean ± SD) and light microscopic observation results on 40 environmental samples.

**Additional file 5: Figure S4.** Summary of the three successive steps of the study and their respective outcomes.

**Additional file 6: Table S2.** Details of cost evaluation.

## Data Availability

The datasets used and/or analysed during the current study are available from the corresponding author on reasonable request.

## Abbreviations

NED: No egg disruption
PK: Proteinase K
TD: Thermal disruption
FPA: FastPrep^®^ with matrix A beads
FPD: FastPrep^®^ with matrix D beads
TD–FPD: TD followed by FPD
TD–FPD-PK: TD-FPD followed by PK

## References

[1] Chen J, Zhou DH, Nisbet AJ, Xu MJ, Huang SY, Li MW (2012). Advances in molecular identification, taxonomy, genetic variation and diagnosis of *Toxocara* spp. Infect Genet Evol.

[2] Holland CV (2017). Knowledge gaps in the epidemiology of *Toxocara*: the enigma remains. Parasitology.

[3] Chen J, Liu Q, Liu GH, Zheng WB, Hong SJ, Sugiyama H (2018). Toxocariasis: a silent threat with a progressive public health impact. Infect Dis Poverty.

[4] Macpherson CN (2013). The epidemiology and public health importance of toxocariasis: a zoonosis of global importance. Int J Parasitol.

[5] Overgaauw PA (1997). Aspects of *Toxocara* epidemiology: human toxocarosis. Crit Rev Microbiol.

[6] Durant JF, Irenge LM, Fogt-Wyrwas R, Dumont C, Doucet JP, Mignon B (2012). Duplex quantitative real-time PCR assay for the detection and discrimination of the eggs of *Toxocara canis* and *Toxocara cati* (Nematoda, Ascaridoidea) in soil and fecal samples. Parasit Vectors.

[7] Tyungu DL, McCormick D, Lau CL, Chang M, Murphy JR, Hotez PJ (2020). *Toxocara* species environmental contamination of public spaces in New York City. PLoS Negl Trop Dis.

[8] Amoah ID, Singh G, Stenstrom TA, Reddy P (2017). Detection and quantification of soil-transmitted helminths in environmental samples: a review of current state-of-the-art and future perspectives. Acta Trop.

[9] Umhang G, Bastien M, Renault C, Faisse M, Caillot C, Boucher JM (2017). A flotation/sieving method to detect *Echinococcus multilocularis* and *Toxocara* spp. eggs in soil by real-time PCR. Parasite.

[10] Mizgajska H (2001). Eggs of *Toxocara* spp. in the environment and their public health implications. J Helminthol.

[11] Mizgajska-Wiktor H (2005). Recommended method for recovery of *Toxocara* and other geohelminth eggs from soil. Wiad Parazytol.

[12] Pabinger S, Rodiger S, Kriegner A, Vierlinger K, Weinhausel A (2014). A survey of tools for the analysis of quantitative PCR (qPCR) data. Biomol Detect Quantif.

[13] Armbruster DA, Pry T (2008). Limit of blank, limit of detection and limit of quantitation. Clin Biochem Rev.

[14] Phasuk N, Kache R, Thongtup K, Boonmuang S, Punsawad C (2020). Soil contamination with *Toxocara* eggs in public schools in rural areas of southern Thailand. J Trop Med.

[15] Azam D, Ukpai OM, Said A, Abd-Allah GA, Morgan ER (2012). Temperature and the development and survival of infective *Toxocara canis* larvae. Parasitol Res.

[16] Schrader C, Schielke A, Ellerbroek L, Johne R (2012). PCR inhibitors—occurrence, properties and removal. J Appl Microbiol.

