# Supplementary figures and images for: Optimized DNA-based identification of Toxocara spp. eggs in soil and sand samples
Source: Parasit Vectors. 2021 Aug 26;14:426. doi: 10.1186/s13071-021-04904-1 (PMC8390219; doi:10.1186/s13071-021-04904-1)

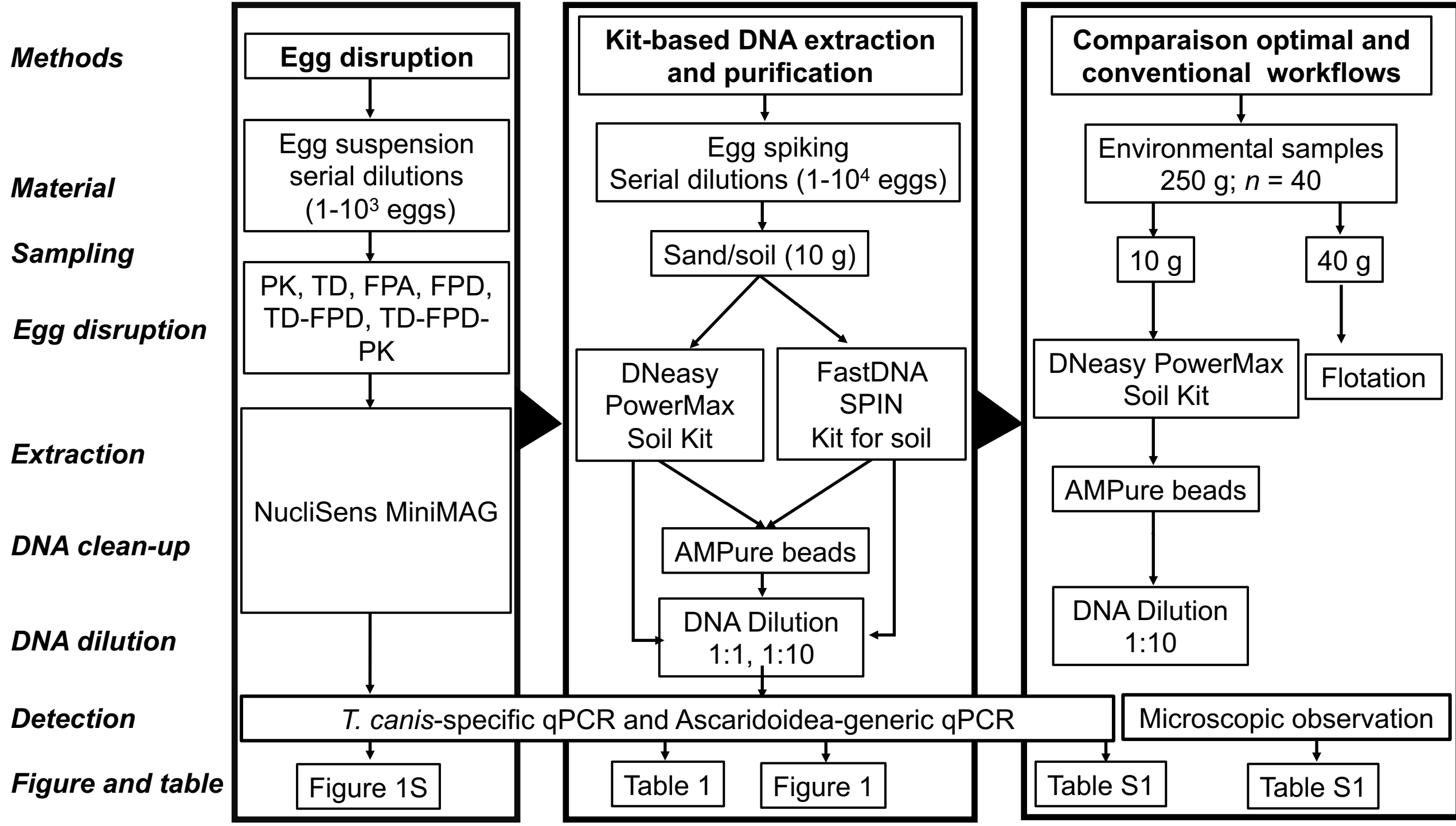

Supplement: Supplementary file 1 — Additional file 1: Figure S1. Stepwise optimisation of the analytical workflow for improving the DNA-based identification of Toxocara spp. eggs. [file 13071_2021_4904_MOESM1_ESM.pdf]

NED

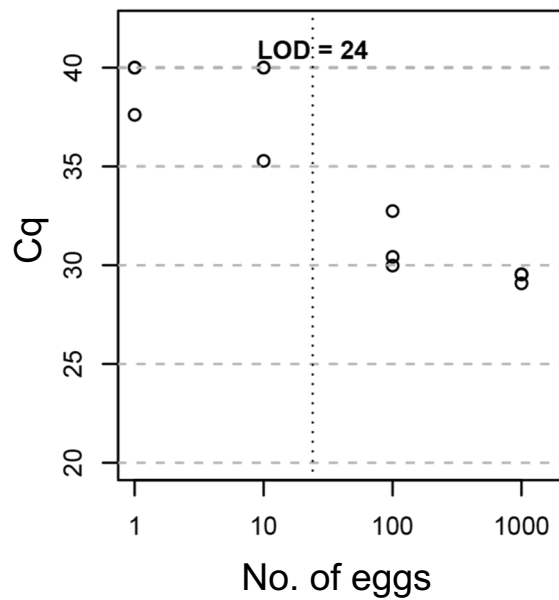

PK

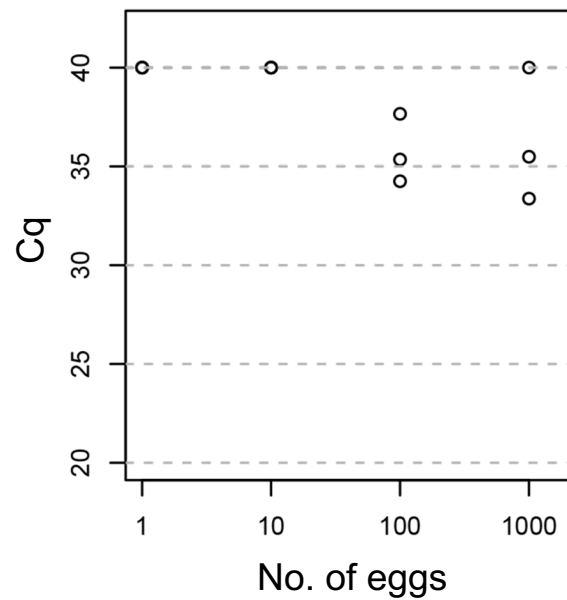

TD

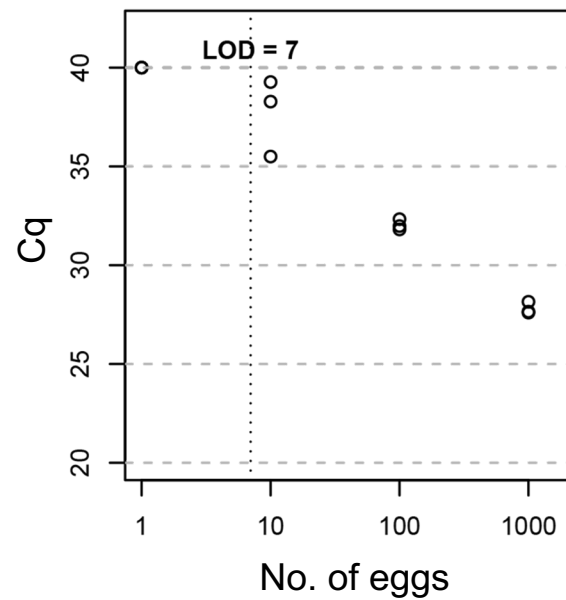

FPA

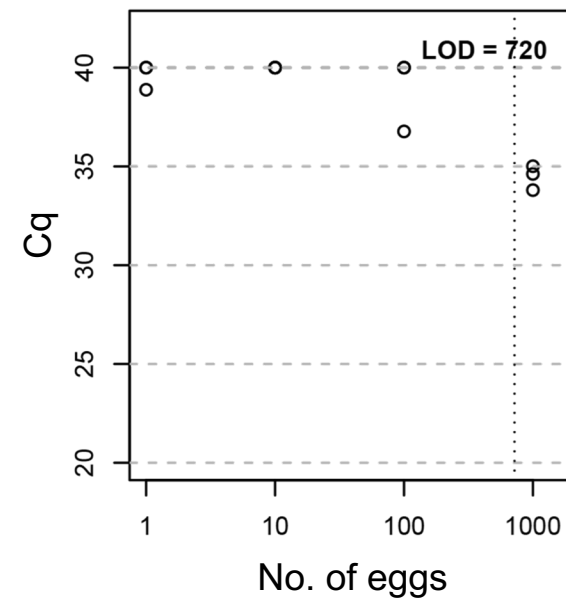

FPD

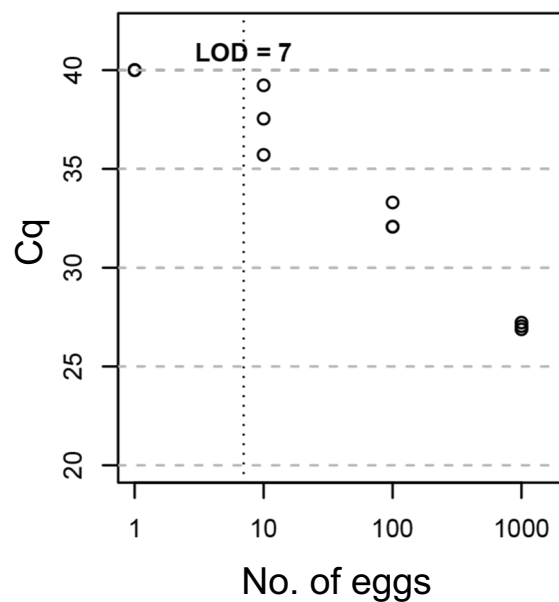

TD-FPD-PK

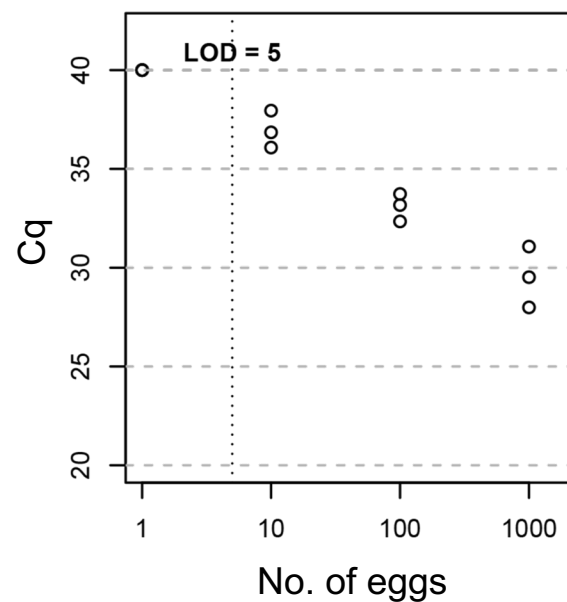

TD-FPD

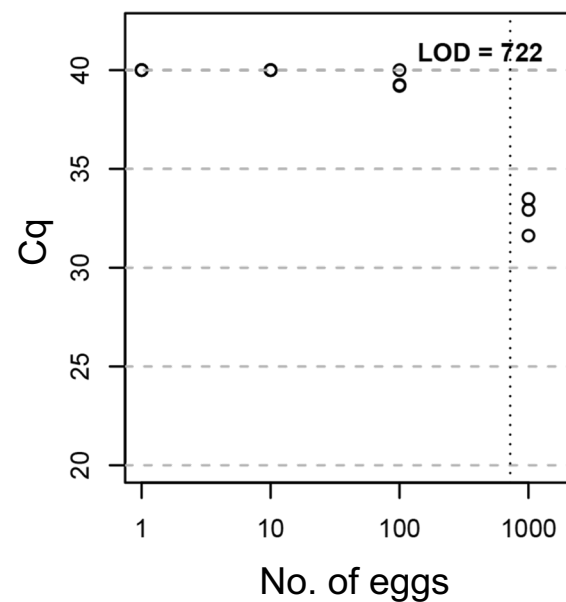

Supplement: Supplementary file 2 — Additional file 2: Figure S2. Comparison of six egg disruption methods and a control (no egg disruption): disruption efficiency is expressed by qPCR Cq values. [file 13071_2021_4904_MOESM2_ESM.pdf]

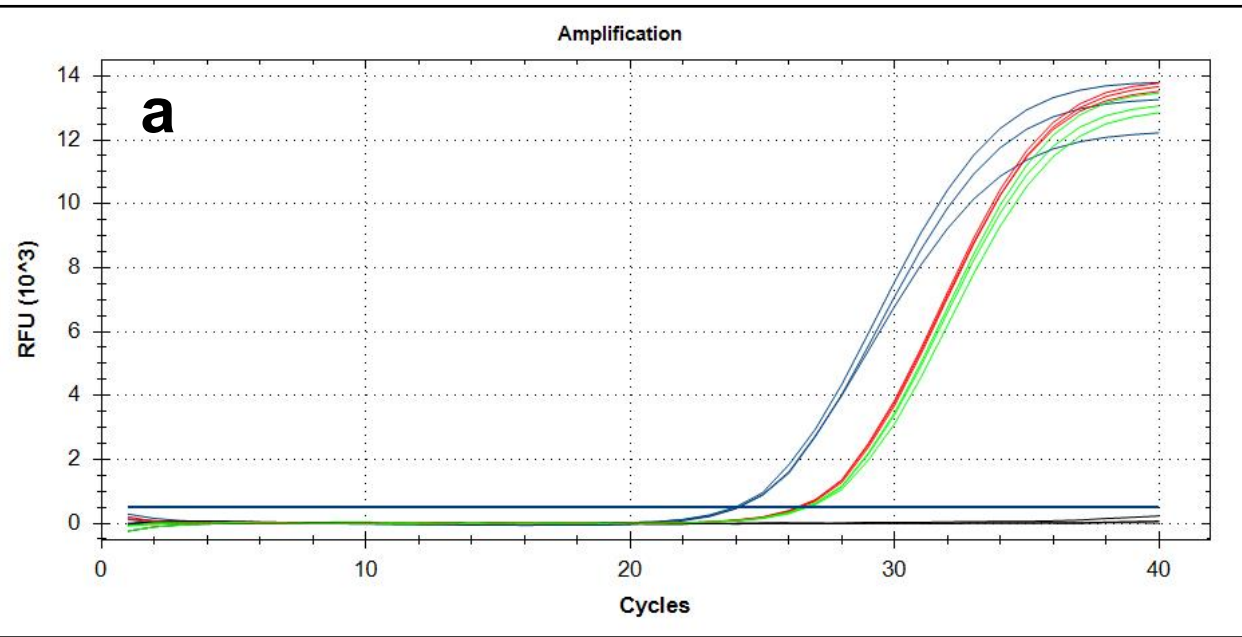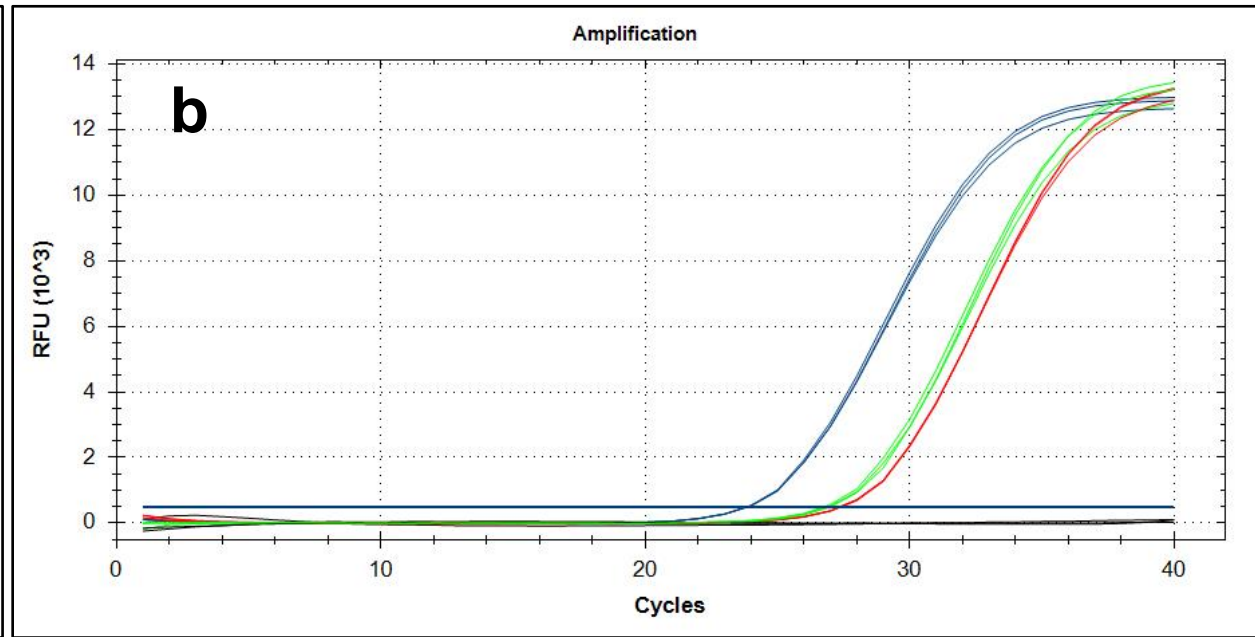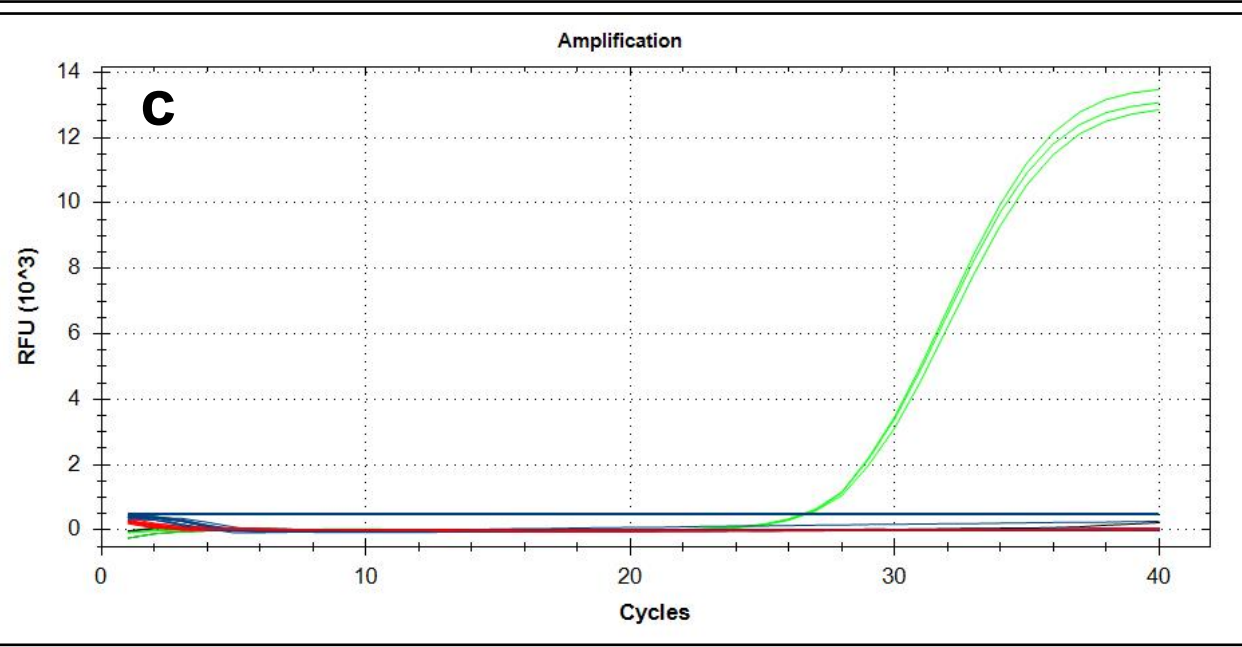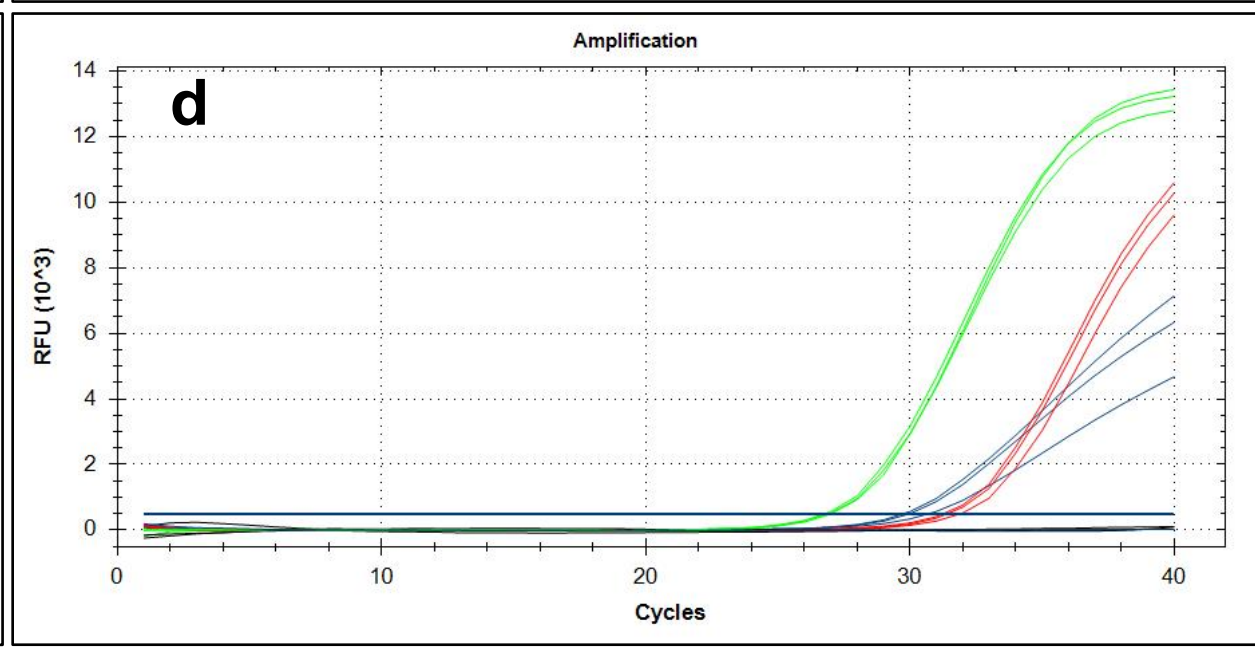

Supplement: Supplementary file 3 — Additional file 3: Figure S3. Examples of qPCR amplification curves according to sample types (sand, soil). qPCR amplification curves obtained after extraction of 104 eggs spiked in sand (a, b) and soil samples (c, d) using the DNeasy® PowerMax® Soil kit. a, c qPCR curves generated without clean-up. b, d qPCR curves generated after purification (AMPure®). The qPCR amplification curves obtained with the Toxocara canis DNA positive control, non-template control (NTC), undiluted DNA and diluted (1:10) DNA are represented in green, black, blue and red, respectively. [file 13071_2021_4904_MOESM3_ESM.pdf]
