# Supplementary material for: Optimized DNA-based identification of Toxocara spp. eggs in soil and sand samples
Source: Parasit Vectors. 2021 Aug 26;14:426. doi: 10.1186/s13071-021-04904-1 (PMC8390219; doi:10.1186/s13071-021-04904-1)
Supplement: Supplementary file 4 — Additional file 4: Table S1. qPCR Cq values (mean ± SD) and light microscopic observation results on 40 environmental samples. [file 13071_2021_4904_MOESM4_ESM.pdf]

Additional file 4: Table S1. qPCR Cq values (mean ± SD) and light microscopic observation results on 40 environmental samples

Sample number	Type	qPCR	Microscopic observation
1	Playground	Negative	Negative
2	Playground	Negative	Negative
3	Playground	34.33 ± 0.51	Negative
4	Playground	Negative	Negative
5	Playground	Negative	Negative
6	Playground	Negative	Negative
7	Playground	35.99 ± 0.68	Negative
8	Playground	33.41 ± 0.55	Negative
9	Playground	Negative	Negative
10	Playground	Negative	Negative
11	Playground	Negative	Negative
12	Playground	Negative	Negative
13	Playground	Negative	Negative
14	Playground	Negative	1 *T. canis* egg with a larva
15	Playground	Negative	Negative
16	Playground	Negative	Negative
17	Playground	33.73 ± 1.04	Negative
18	Playground	Negative	Negative
19	Playground	Negative	Negative
20	Playground	Negative	Negative
21	Backyard	Negative	Negative
22	Backyard	Negative	Negative
23	Backyard	Negative	Negative
24	Backyard	Negative	Negative
25	Backyard	Negative	Negative
26	Backyard	Negative	Negative
27	Backyard	Negative	Negative
28	Backyard	Negative	Negative
29	Backyard	Negative	Negative
30	Backyard	Negative	Negative
31	Backyard	Negative	Negative
32	Backyard	24.30 ± 0.09	7 *T. canis* eggs with a larva
33	Backyard	30.55 ± 0.23	Negative
34	Backyard	Negative	Negative
35	Backyard	Negative	Negative
36	Backyard	29.72 ± 0.04	1 *T. canis* egg
37	Backyard	23.30 ± 0.31	12 *T. canis* eggs with a larva
38	Backyard	Negative	Negative
39	Backyard	Negative	Negative
40	Backyard	Negative	Negative

"
