# Supplementary material for: Optimized DNA-based identification of Toxocara spp. eggs in soil and sand samples
Source: Parasit Vectors. 2021 Aug 26;14:426. doi: 10.1186/s13071-021-04904-1 (PMC8390219; doi:10.1186/s13071-021-04904-1)
Supplement: Supplementary file 5 — Additional file 5: Figure S4. Summary of the three successive steps of the study and their respective outcomes. [file 13071_2021_4904_MOESM5_ESM.pdf]

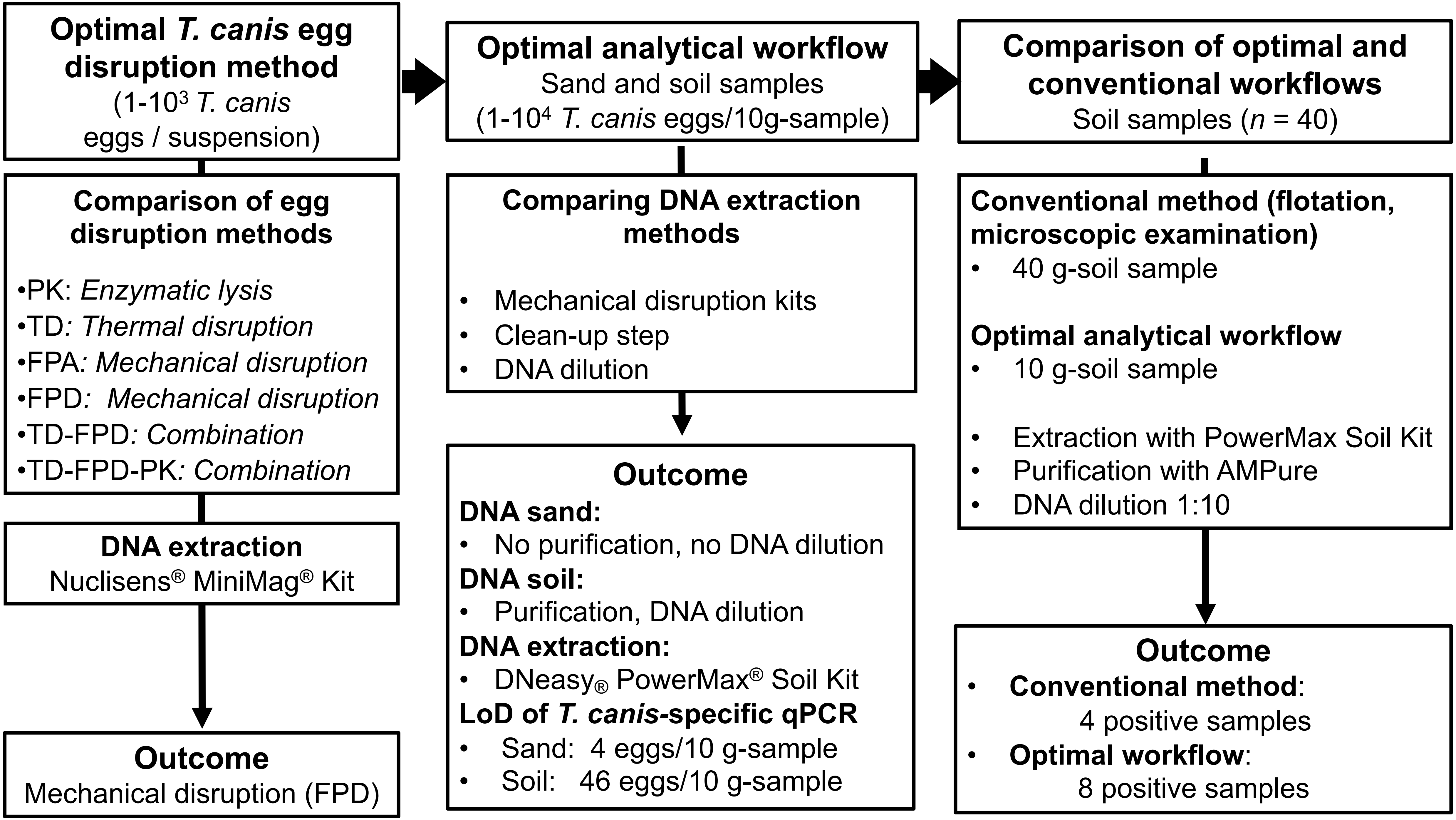
Optimal T. canis egg disruption method
(1-10^3 T. canis eggs / suspension)
Comparison of egg disruption methods
•PK: Enzymatic lysis
•TD: Thermal disruption
•FPA: Mechanical disruption
•FPD: Mechanical disruption
•TD-FPD: Combination
•TD-FPD-PK: Combination
DNA extraction
Nuclisens® MiniMag® Kit
Outcome
Mechanical disruption (FPD)
Optimal analytical workflow
Sand and soil samples
(1-10^4 T. canis eggs/10g-sample)
Comparing DNA extraction methods
• Mechanical disruption kits
• Clean-up step
• DNA dilution
Outcome
DNA sand:
• No purification, no DNA dilution
DNA soil:
• Purification, DNA dilution
DNA extraction:
• DNeasy® PowerMax® Soil Kit
LoD of T. canis-specific qPCR
• Sand: 4 eggs/10 g-sample
• Soil: 46 eggs/10 g-sample
Comparison of optimal and conventional workflows
Soil samples (n = 40)
Conventional method (flotation, microscopic examination)
• 40 g-soil sample
Optimal analytical workflow
• 10 g-soil sample
• Extraction with PowerMax Soil Kit
• Purification with AMPure
• DNA dilution 1:10
Outcome
• Conventional method: 4 positive samples
• Optimal workflow: 8 positive samples
