# Supplementary material for: Optimized DNA-based identification of Toxocara spp. eggs in soil and sand samples
Source: Parasit Vectors. 2021 Aug 26;14:426. doi: 10.1186/s13071-021-04904-1 (PMC8390219; doi:10.1186/s13071-021-04904-1)
Supplement: Supplementary file 6 — Additional file 6: Table S2. Details of cost evaluation. [file 13071_2021_4904_MOESM6_ESM.pdf]

Additional file 6: Table S2. Details of cost evaluation.

Cost assessment according to optimal analytical workflow	DNeasy® PowerMax® Soil kit	FastDNA™ SPIN Kit for soil
Consumables (tips, multi-well plate, free water, ...)	53,11 €	
Extraction step	349,00 €	227,00 €
Clean-up	175,29 €	
qPCR reagents	65,04 €	
Total cost for 10 samples (€, excl. taxes)	642,44 €	520,44 €
